# SiMYB3 in Foxtail Millet (*Setaria italica*) Confers Tolerance to Low-Nitrogen Stress by Regulating Root Growth in Transgenic Plants

**DOI:** 10.3390/ijms20225741

**Published:** 2019-11-15

**Authors:** Linhao Ge, Yining Dou, Maomao Li, Pengju Qu, Zhang He, Y Liu, Zhaoshi Xu, Jun Chen, Ming Chen, Youzhi Ma

**Affiliations:** 1 National Key Facility for Crop Genetic Resources and Genetic Improvement, Key Laboratory of Crop Genetics and Breeding, Ministry of Agriculture/Institute of Crop Sciences, Chinese Academy of Agricultural Sciences, Beijing 100081, China; beatsgg@163.com (L.G.); douyining12@163.com (Y.D.); 13610642179@163.com (P.Q.); hz384298890@163.com (Z.H.); xuzhaoshi@caas.cn (Z.X.); chenjun01@caas.cn (J.C.); 2 Rice Research Institute, Jiangxi Academy of Agricultural Sciences/ Rice National Engineering Laboratory, Nanchang 330200, China; lmm3056@163.com; 3 Xiangyang Academy of Agricultural Sciences, Xiangyang 441057, China; myseaky_2007@163.com

**Keywords:** foxtail millet, low nitrogen stress, transcriptome analysis, MYB-like transcription factor, transgenic plants

## Abstract

Foxtail millet (*Setaria italica*), which originated in China, has a strong tolerance to low nutrition stresses. However, the mechanism of foxtail millet tolerance to low-nitrogen stress is still unknown. In this study, the transcriptome of foxtail millet under low-nitrogen stress was systematically analyzed. Expression of 1891 genes was altered, including 1318 up-regulated genes and 573 down-regulated genes. KEGG (Kyoto Encyclopedia of Genes and Genomes) analysis revealed that 3% of these genes were involved in membrane transport and 5% were involved in redox processes. There were 74 total transcription factor (TF) genes in the DEGs (differentially expressed genes), and MYB-like transcription factors accounted for one-third (25) of the TF genes. We systematically analyzed the characteristics, expression patterns, chromosome locations, and protein structures of 25 MYB-like genes. The analysis of gene function showed that *Arabidopsis* and rice overexpressing *SiMYB3* had better root development than WT under low-nitrogen stress. Moreover, EMSA results showed that *SiMYB3* protein could specifically bind MYB elements in the promoter region of *TAR2*, an auxin synthesis related gene and MYB3-TAR2 regulate pair conserved in rice and foxtail millet. These results suggested that *SiMYB3* can regulate root development by regulating plant root auxin synthesis under low-nitrogen conditions.

## 1. Introduction

Nitrogen is an essential nutrient for plant growth [1]. Because of the high demand for nitrogen, plant growth and development are often limited by insufficient nitrogen supply [2,3]. The root is the main organ by which plants obtain nitrogen from soil, and morphological and physiological characteristics of the root are important for effective nitrogen use in plants. Changes in root structure have allowed plants to develop functions that improve their adaptability to various environments [4,5,6,7]. Studies have shown that many mineral nutrients affect root development by affecting the biosynthesis, transport, and signal transduction of plant hormones [8,9,10]. 

Low nitrate stress treatment can induce biosynthesis of auxin in the root [11,12,13]. In addition to the transport of auxin, the signaling transduction of the auxin hormone in the root is affected by low-nitrogen stress. The auxin-resistant mutant *axr4* shows no root developmental changes when it lacks nitrate, which provides evidence of overlap between auxin and nitrate signaling pathways [14]. Plants have many active auxins, and IAA is the most thoroughly studied natural auxin [15]. Two major pathways for IAA biosynthesis have been proposed: the tryptophan (Trp)-independent and Trp-dependent pathways. In Trp-dependent IAA biosynthesis, four pathways have been postulated in plants: (i) the indole-3-acetamide (IAM) pathway; (ii) the indole-3-pyruvic acid (IPA) pathway; (iii) the tryptamine (TAM) pathway; and (iv) the indole-3-acetaldoxime (IAOX) pathway [16]. Trp aminotransferase *TAA1* and its close homologs *TAR1* and *TAR2* convert L-Trp to IPyA [17,18]. Multiple lines of evidence suggest that the IPyA pathway is the primary mechanism for de novo synthesis of auxin in plants [19]. Mutations in *TAR2* result in decreased lateral root development under low-nitrogen conditions and low *N*-stimulated lateral root development is dependent on auxin, which is involved in root synthesis by *TAR2* [20].

Transcription factors (TFs) play an extremely important role in plants by regulating the expression of stress-related genes, which regulates growth and adaptation to environmental changes [21,22,23]. The MYB TFs are widely involved in secondary metabolic pathways (including anthocyanin pathways), growth, signal transduction, and disease resistance pathways in plants [24]. Based on the number and location of MYB domains, MYB is divided into four main groups: 1R (R1/2, R3-MYB), 2R (R2R3-MYB), 3R (R1R2R3-MYB), and 4R, which contain four R1/R2-like repeats [25]. R2R3-like MYBs are involved in phenylpropanoid metabolism, the anthocyanin biosynthetic pathway, and lignin synthesis in many plants [26,27,28,29,30]. R2R3-MYBs are also involved in a variety of phytohormone signaling pathways, like salicylic acid [31], abscisic acid [32], gibberellic acid [33], and jasmonic acid [34]. MYB TFs are also involved in the regulation of abiotic stresses such as nutrient starvation in plants, and the MYB-like TFs are involved in flavonol synthesis, which is partially affected by nitrogen deficiency [35]. The R2R3-MYB-like TFs control not only the regulation of flavonoid biosynthesis, but also the regulation of epidermal cell differentiation and cell patterning in root hair development [24,36]. Low-nitrogen-responsive MYB members, such as *ATR1* and *ATR2*, are involved in higher plant regulation under conditions of nitrogen deficiency [37,38].

Foxtail millet (*Setaria italica*) is one of the oldest crops in the world, and it is believed that cultivation may have begun in Gansu, northwestern China, in 5900 BP [39]. Foxtail millet grows in arid soils and does not require high levels of nitrogen fertilizers. This suggests that foxtail millet has a high tolerance for low-nitrogen stress, which is advantageous for food production in developing countries [40]. Its drought-resistant properties and low-nitrogen stresses, along with its small diploid genome (~510 Mb) [41,42], make foxtail millet an ideal model species to study drought response and low-nitrogen stresses in monocotyledons [43,44]. Additionally, foxtail millet root systems are generally strong, which could be related to its strong resistance to drought and low-nitrogen stresses [45,46]. Recently, functional genomics research of foxtail millet has begun, but the mechanism of strong stress resistance of foxtail millet, especially the relationship between stress resistance and the root development of millet, is still largely unknown.

In this study, we analyzed the transcriptome of foxtail millet under low-nitrogen stress through RNA-seq. We found that under low-nitrogen stress, the number of MYB TFs was the highest compared to the other TF families. Based on gene function analysis, MYB-like TF *SiMYB3* significantly improved the low-nitrogen tolerance of transgenic plants grown on media and under field conditions. At the same time, *SiMYB3* affected the root development of transgenic plants by regulating the expression of auxin synthesis-related gene *TAR2*, which may be the mechanism by which *SiMYB3* improves plant tolerance to low-nitrogen stress. This study has increased our understanding of the stress-resistant regulatory network of foxtail millet.

## 2. Results

### 2.1. RNA-Seq Analyses of Low-Nitrogen-Induced Foxtail Millet

To obtain a global view of transcriptome profiles relevant to the *N*-deficient treatment in Chinese foxtail millet Longgu 25, the high throughput RNA-Seq analyses on poly(A^+^)-enriched RNAs from the CK and Low Nitrogen treatment (LN) libraries were performed using the Solexa/Illumina platform. The results of transcriptome analysis showed that under low-nitrogen stress, there were 1889 differentially expressed genes (DEG) including 1317 up-regulated genes and 572 down-regulated genes (log_2_ ≥ 1 or log_2_ ≤ −1) (Supplementary Table S1). These DEGs were enriched in different GO terms by three classification methods, including biological processes (319 GO terms) (Supplementary Table S2), cell components (65 GO terms) (Supplementary Table S3), and molecular functions (187 GO terms) (Supplementary Table S4). Some of the GO terms are shown in Figure S1A and some GO terms, such as response to stimulus (biological process) and antioxidant activity (molecular function), were related to the stress response. To identify the most important biochemical and metabolic pathways related to the DEGs, we performed a KEGG analysis (Figure S1B). The results showed that 1103 DEGs were enriched in 113 pathways and most DEGs were involved in four main pathways, including metabolic pathway (303 genes), biosynthesis of secondary metabolites (251 genes), plant-pathogen interaction (163 genes), and plant hormone signal transduction (107) (Figure S1B). DEGs were involved in different processes, including protein phosphorylation (4%), transmembrane transport (3%), and the oxidation reduction process (5%) in foxtail millet (Figure S1C).

**Figure 1:**
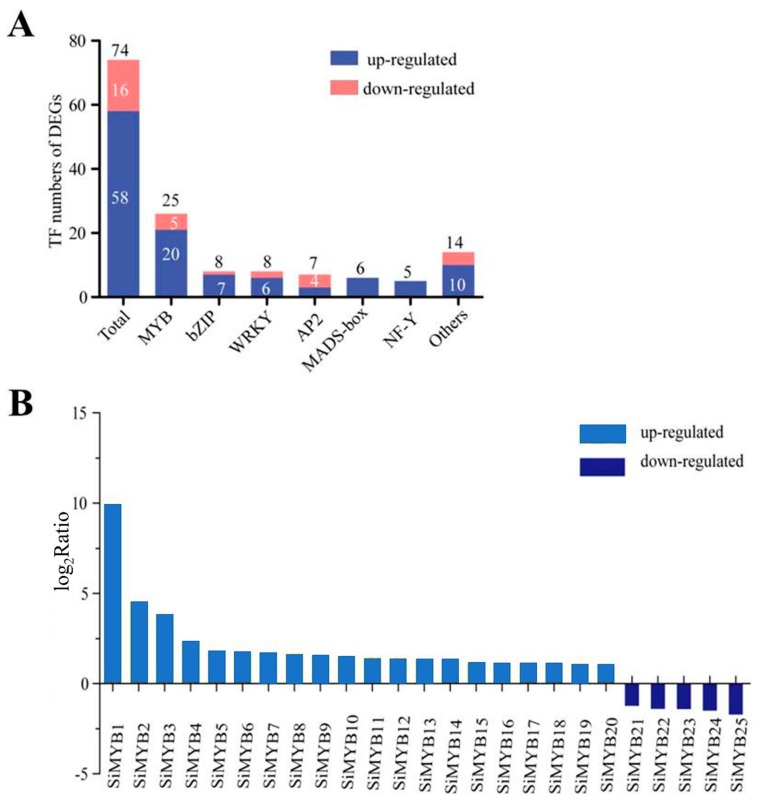
RNA-seq analysis of low-nitrogen treatment of four-leaf stage foxtail millet. (**A**) Transcription factor gene classification of DEGs. There were 74 transcription factors in 1889 DEGs. (**B**) Differential expression fold analysis of SiMYB genes in response to low-nitrogen stress in foxtail millet. The value of the Y-axis represents the log_2_Ratio.

Because transcription factors (TFs) play an important role in regulating plant stress response, we selected TF genes from DEGs under low-nitrogen treatment for further analysis. The results showed that a total of 74 TFs (58 upregulated, 16 downregulated) were involved in low-nitrogen stress response in foxtail millet. These 74 TF genes were from different gene families, including 25 MYB (20 up, 5 down), 8 bZIP (7 up, 1 down), 8 WRKY (6 up, 2 down), 7 AP2 (3 up, 4 down), 6 MADS-box (6 up, 0 down), 5 NF-Y (5 up, 0 down), and 24 other (10 up, 14 down) (Figure 1A,B). In those gene families, the number of TFs from the MYB gene family was the highest, suggesting that MYB-like genes play more important roles in the response to low-nitrogen stress in foxtail millet. MYB proteins represent one of the largest transcription factor families in plants, and all 209 *S. italica* MYB (SiMYB) genes were physically mapped onto nine chromosomes of foxtail millet [47]. Based on these results, we focused on the MYB gene family for further analyses.

### 2.2. Characteristics Analysis of 25 MYB-Like Transcription Factors in Response to Low-Nitrogen Stress in Foxtail Millet

**Figure 2:**
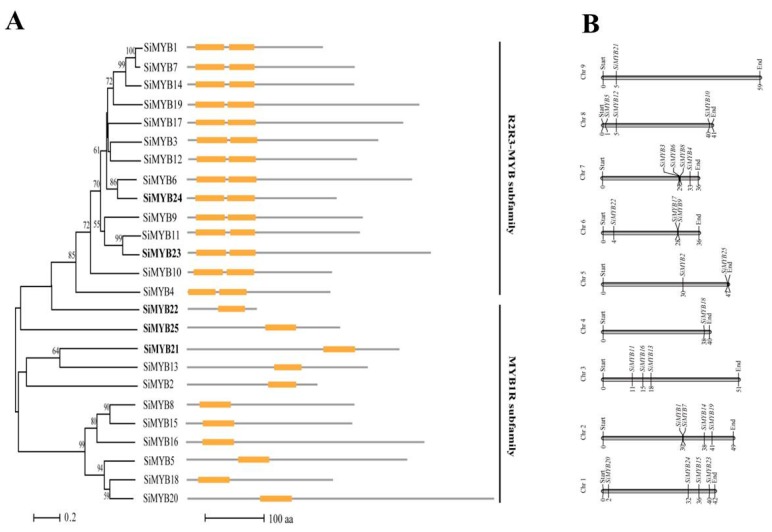
Phylogenetic and protein structure analysis of the *SiMYBs* gene family in foxtail millet. (**A**) Phylogenetic tree of SiMYBs gene family in foxtail millet. Bold names represent downregulated genes. Protein structure of SiMYBs gene family in foxtail millet. The yellow box represents DNA binding domain. (**B**) Chromosomal localization of the SiMYBs gene family in foxtail millet. Phylogenetic trees were constructed using MEGA 6.0 with the neighbor-joining method [48] and 1000 bootstrap replications.

We named the 25 MYB TFs SiMYB1 as SiMYB25 based on high to low gene expression levels under low-nitrogen conditions (Figure 1B). Given that the genes of the MYB family were systematically analyzed and named [47], we have shown the names associated with them in Supplementary Table S5 (the biochemical characteristics of 25 MYB TFs are displayed). The nucleotide sequence lengths of 25 MYB-like TFs varied greatly from 774 to 4764 bp; the amino acid sequence length of the MYB TF members varied between 116 and 507 amino acids. Through the prediction of protein domains, 25 MYB-like proteins contained a conserved DNA binding domain (Pfam PF00249) and at least one helix-turn-helix (HTH) domain. In order to analyze the evolutionary relationships of these 25 MYB genes within the gene family, phylogenetic trees were constructed based on multiple sequence alignments (Figure 2A). The results showed that the MYB-like protein family was divided into two subfamilies, including MYB1R and R2R3-MYB (Figure 2A). The protein structure analysis showed that the MYB1R subfamily protein contained only one HTH domain and the R2R3-MYB subfamily contained two HTH domains (Figure 2A). The loci of different chromosomes of 25 SiMYB-like genes are shown in Figure 2B.

### 2.3. Subcellular Localization, Expression Pattern, and Gene Functions of SiMYB3

**Figure 3:**
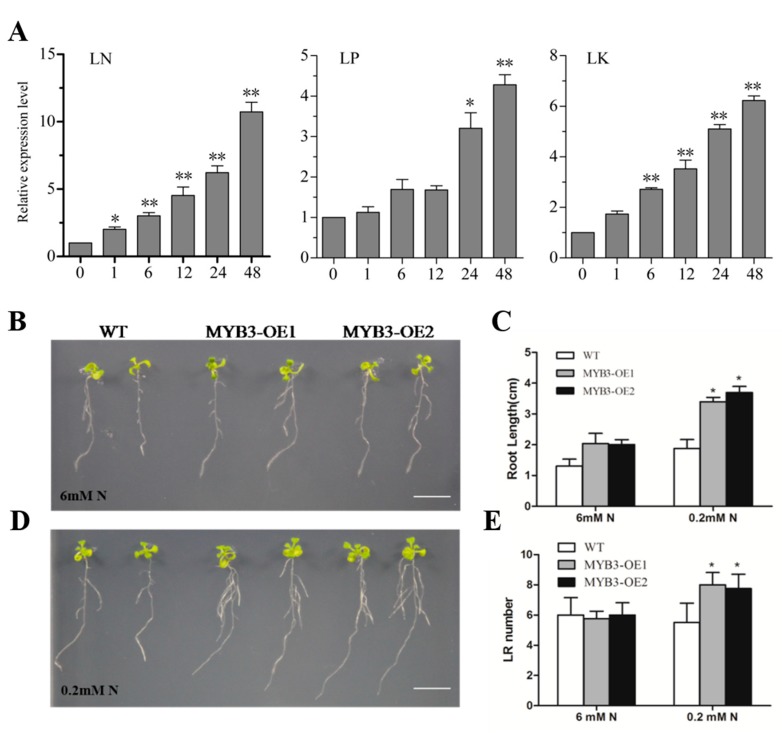
Expression pattern analysis of SiMYB3, and over-expression of SiMYB3 in *Arabidopsis* increased the root growth of seeds under low-nitrogen treatment. (**A**) Expression profile analysis of SiMYB3 under Low Nitrogen (LN), Low Phosphorus (LP), and Low Potassium (LK) treatment conditions. Bar = 5 μm. B-E: Seeds were germinated on 1/2 MS (Nitrogen free) medium supplemented with different concentrations of Nitrogen (0.2 and 6 mM). (**B**) Control (6 mM N); (**C**) measurements of total root length; (**D**) LN treatment (0.2 mM N); (**E**) measurements of number of lateral roots. Bar = 1 cm. Each data point is the mean (±SE) of three experiments. Statistical significance was determined using Student’s t-tests (* *p* < 0.05, ** *p* < 0.01). To identify gene function, we transformed *SiMYB3* into dicotyledonous model plants (*Arabidopsis*) and monocotyledonous model plants (rice). The expression of *SiMYB3* in transgenic plants was verified by RT-PCR (Figure S2).

Since genetic transformation of foxtail millet has been unsuccessful, we transformed some MYB members into *Arabidopsis* and rice to identify the gene functions of the 25 MYB TFs. Our results indicated that overexpression of SiMYB3 promoted root development under low-nitrogen conditions in transgenic *Arabidopsis* (Figure 3). Therefore, we chose SiMYB3 for further analysis and have only reported the results of the study on *SiMYB3* in this paper.

Expression of *SiMYB3* was induced by different low nutrient stresses, including low-nitrogen, low phosphorus, and low potassium. Among these, nutrient stress-induced expression of *SiMYB3* under low-nitrogen stress was the highest, and the expression of *SiMYB3* was the highest (11 times higher than untreated) at 48 h after treatment (Figure 3A). Of the 25 low-nitrogen responsive MYB TFs, the inducible expression levels of *SiMYB3* ranked third. Phylogenetic trees showed that SiMYB3 was in the R2R3-MYB subfamily and was most closely related to SiMYB12 (Figure 2A). Gene mapping analysis showed that *SiMYB3* was located on the seventh chromosome of foxtail millet, closest to *SiMYB6* and *SiMYB8* (Figure 2B).

In *Arabidopsis,* the results of gene function analysis under low-nitrogen stress showed that after 10 days, total root length and lateral root number of WT and transgenic plants were similar under normal conditions (6 mM nitrogen). In contrast, under low-nitrogen treatment (0.2 mM nitrogen), the total root length of transgenic lines OE1 and OE2 was significantly longer than those of WT, and the number of lateral roots in transgenic plants was higher than WT (Figure 3B–E). These results indicated that overexpression of *SiMYB3* conferred tolerance to low nitrogen in transgenic *Arabidopsis*. 

**Figure 4:**
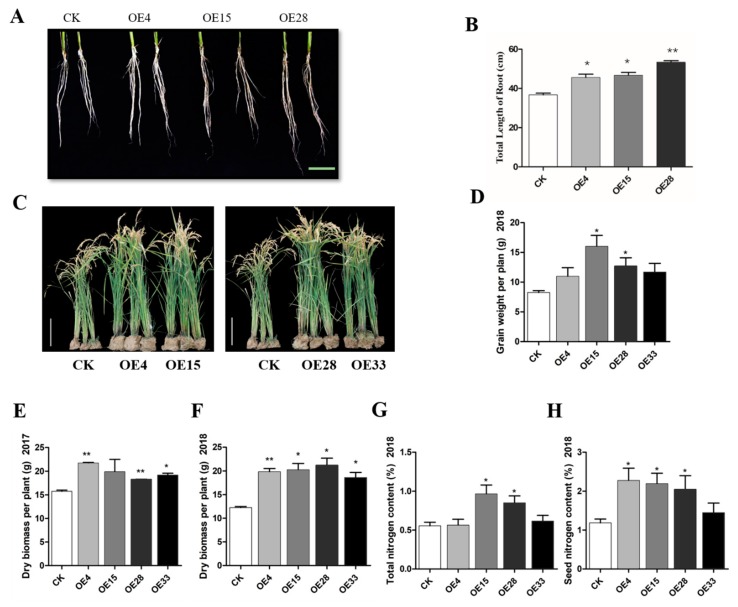
Phenotype of SiMYB3 transgenic lines in rice. (**A**) Phenotype of SiMYB3 transgenic lines under Hoagland’s 0.2 mM nitrogen liquid medium. (**B**) Measurements of total root length; Bar = 5 cm. C-H: phenotype analysis of rice *SiMYB3*-OE lines under no nitrogen fertilizer in the field. (**C**) Phenotype analysis of *SiMYB3* transgenic lines under no nitrogen fertilizer in the field, (**D**) grain weight per plant, (**E,F**) dry weight per plant in 2017 and 2018, total nitrogen content (**G**), and seed nitrogen content (**H**). Bar = 10 cm. Values are the mean ± SE of at least three replicate experiments. The statistical significance was determined using Student’s *t*-tests. Asterisks indicate the significant difference between *SiMYB3* transgenic lines and WT plants (* *p* < 0.05, ** *p* < 0.01).

To test gene function in a monocotyledonous crop, we transformed *SiMYB3* into rice after identifying transgenic rice lines. Under the low-nitrogen treatment (0.2 mM nitrogen), the total root lengths of transgenic rice lines OE4, OE15, and OE28 were significantly longer than those of CK (Figure 4A,B). We completed the tolerance analysis of low-nitrogen stress in the whole growth period of rice in the field for two years in Nanchang City, Jiangxi Province. After two years of study, our results showed that the dry biomass in transgenic lines OE4, OE28, and OE33 were significantly higher than WT (Figure 4E,F). The grain weight, total nitrogen content, and seed nitrogen content of plants all increased in transgenic rice lines when compared to WT (Figure 4).

These results indicated that overexpression of *SiMYB3* in transgenic *Arabidopsis* and rice promoted root growth and increased grain weight under low-nitrogen conditions, suggesting that *SiMYB3* conferred tolerance to nitrogen stress in transgenic plants.

### 2.4. SiMYB3 Enhanced Tolerance to Low-Nitrogen Stress by Regulating Auxin Synthesis-Related Genes, TAR2, and Low-Nitrogen Stress-Related Genes

**Figure 5:**
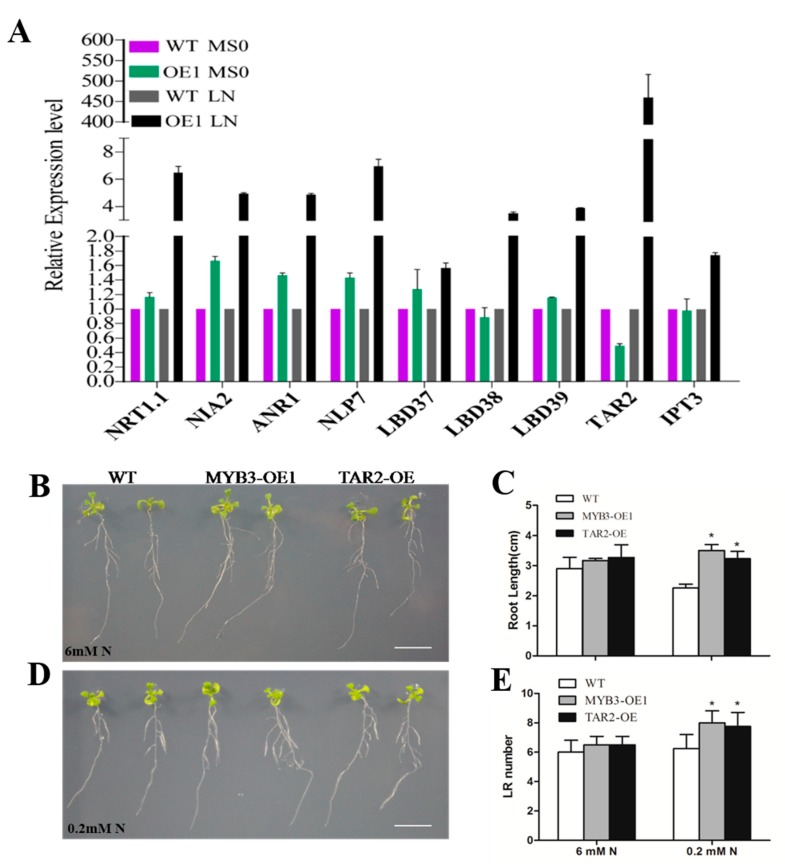
Phenotype analysis of *SiMYB3* and *TAR2* overexpression line under different conditions. B-E: Seeds were germinated on 1/2 MS (Nitrogen free) medium supplemented with different concentrations of Nitrogen (0.2 and 6 mM). (**A**) Levels of gene expression in response to nitrogen under low-nitrogen stress. Fourteen-day-old wild type and overexpression *Arabidopsis* plants were treated with low nitrogen (0.2 mM nitrogen) and samples were harvested after 24 h for RNA extraction. (**B**) Control (6 mM N); (**C**) Measurements of total root length; (**D**) low-nitrogen treatment (0.2 mM N); (**E**) Measurements of number of lateral roots. Bar =1 cm. Values are the mean ± SE of at least three replicate experiments. Statistical significance was determined using Student’s *t*-tests. Asterisks indicate the significant differences between SiMYB3 transgenic lines and WT plants (* *p* < 0.05).

**Figure 6:**
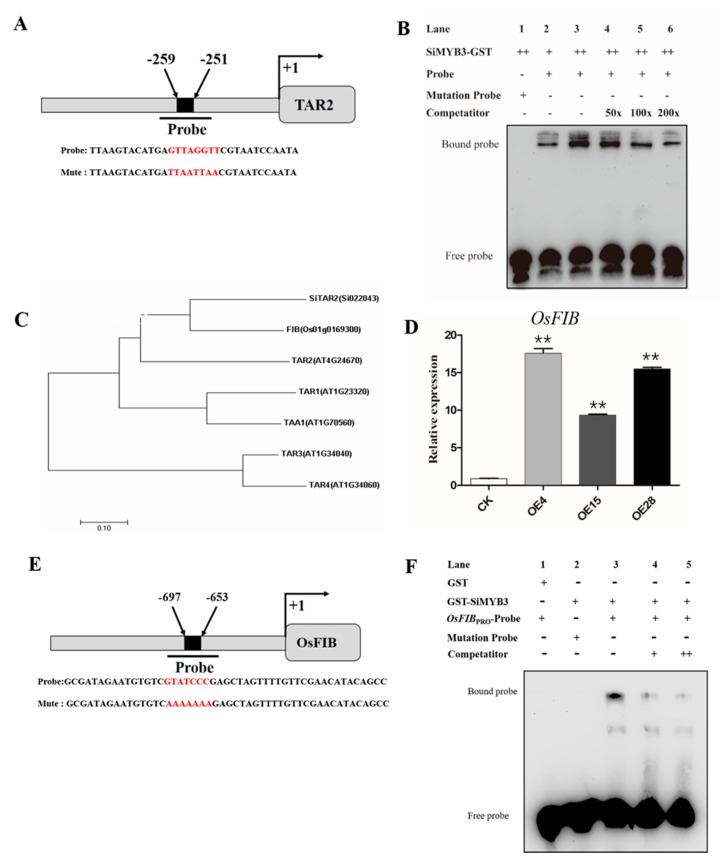
SiMYB3 regulates the expression of the auxin synthesis gene. (**A**) Schematic diagram of the *TAR2* promoter region showing the MYB binding motifs. (**B**) Electrophoretic mobility shift assays (EMSA) indicating SiMYB3 binding specific MYB-like motifs. (**C**) Phylogenetic tree of *TAR2* family members from Arabidopsis, rice, and foxtail millet. (**D**) Differential expression of *OsFIB* gene in rice transgenic lines under low-nitrogen treatment. (**E**) Schematic diagram of *OsFIB* promoter region showing the MYB binding motifs. (**F**) Electrophoretic mobility shift assays (EMSA) indicating SiMYB3 binding specific MYB-like motifs. Values are the mean ± SE of at least three replicate experiments. Statistical significance was determined using Student’s *t*-tests. Asterisks indicate the significant differences between *SiMYB3* transgenic lines and CK plants.

To illustrate the regulation mechanism of SiMYB3 in transgenic plants, we detected the expression of some low-nitrogen stress-related genes. The results showed that many genes, including *NRT1.1*, *NIA2*, *ANR1*, *NLP7*, *LBD37*, *LBD38*, *LBD39*, *TAR2*, and *IPT3,* were highly upregulated in the transgenic *Arabidopsis* (Figure 5A). Among those genes, the induced expression level of *TAR2* was higher than WT. Similarly, we found that the rice auxin synthesis-related gene *OsFIB* [49], which is a homologous gene of *TAR2* in rice, also has a high level of expression in transgenic rice (Figure 6). Our results also showed that the foxtail millet auxin synthesis-related gene *SiTAR2* is upregulated under low-nitrogen stress (Figure S3A).

We then compared phenotypes of two transgenic plants overexpressing *SiMYB3* (OE1) and *TAR2* (*TAR2*-OE) under low-nitrogen stress (Figure 5B–E). The results showed that there was no difference in total root length or lateral root number between WT and OE1 or *TAR2*-OE plants under normal conditions (6 mM nitrogen), but under low-nitrogen stress (0.2 mM nitrogen), the phenotype of OE1 was similar to *TAR2*-OE. The total root length and lateral root number of OE1 and *TAR2*-OE were significantly larger than WT (Figure 5C,E). These results suggested that SiMYB3 and TAR2 play similar roles in *Arabidopsis* during a low-nitrogen stress response. To further identify the binding activity of SiMYB3 on the promoter of *TAR2*, we completed an EMSA assay of SiMYB3 in vitro. We analyzed all cis-elements 2 kb upstream of the *TAR2* gene and found some MYB elements. To confirm the binding specificity, we performed EMSA using recombinant SiMYB3 and a probe designed using an MYB element in the promoter region of *TAR2* (Figure 6A). When the DNA probe was incubated with the SiMYB3 protein, the shifted band was clearly detected (Figure 6B, lanes 2 and 3), but no shifted band was observed when the mutant probe was incubated with SiMYB3 (Figure 6B, lane 1). Additionally, SiMYB3 protein could also bind the promoter region of rice auxin synthesis-related gene *OsFIB* (Figure 6E,F), and millet auxin synthesis-related gene *SiTAR2* (Figure S3).

## 3. Discussion

Foxtail millet has a high tolerance for low-fertility soil [40]. In order to improve the efficiency of fertilizer use in certain crops (particularly Gramineae crops), it is important to clarify how foxtail millet regulates its tolerance to low nutrient stress. To that end, we systematically analyzed the transcriptome of foxtail millet under low-nitrogen treatment. The results showed a total of 1889 DEGs, some of which participated in stress-related GO terms, including the response to stimulus and antioxidant activity. According to the identified pathways, 3% of the genes were involved in transmembrane transport and 5% were involved in the oxidation reduction process (Figure S1C). Among those DEGs, there were 74 TF genes belonging to different gene families, including MYB, bZIP, WRKY, AP2, MADS-box, and NF-Y. MYB-like TFs accounted for one-third of the low-nitrogen response TFs, suggesting that MYB-like TFs play an important role in response to low-nitrogen stress in foxtail millet. In *Arabidopsis*, most nitrogen-related MYB family transcription factors belong to the R2R3-type MYB subfamily [30,37,50]. MYB protein represents one of the largest families of transcription factors in plants and plays an important role in a variety of developmental and stress response processes. Currently, 209 MYB-type transcription factors have been identified in foxtail millet [47]. In this study, we found that MYB-like transcription factors that respond to low-nitrogen stress are from two different subfamilies, the R2R3-type MYB subfamily (14 genes) and the MYB1R subfamily (11 genes). Moreover, SiMYB3 is from the R2R3-type MYB subfamily, suggesting that members in this subfamily are important for tolerance to low-nitrogen stress in monocotyledonous and dicotyledonous plants.

We overexpressed the SiMYB3 gene in *Arabidopsis* and rice. Low-nitrogen experiments at the seedling stage demonstrated that transgenic lines enhanced root development when compared to the wide type (Figure 4 and Figure 5), which suggests that SiMYB3 significantly improved the tolerance of transgenic rice to low-nitrogen stress. In the seedling stage of *Arabidopsis* and rice, no significant differences in biomass were observed aboveground, but through field experiments, we found that transgenic rice has high biomass and yield (Figure 4C–H). We conclude that SiMYB3 helps plant growth by enhancing root development and promoting nitrogen uptake, stimulating growth in a low-nitrogen environment.

When plants face the challenge of environmental change, the response of the roots is often based on auxin, ethylene, and cytokinin. Understanding how hormones and genes interact to coordinate plant growth in a changing environment is a major challenge in plant developmental biology [51]. We found that under low-nitrogen conditions, the expression level of *TAR2* was highly upregulated in the SiMYB3 overexpressing lines (Figure 5A). *TAR2* is an auxin biosynthesis-related gene that is induced under low-nitrogen conditions and is involved in the regulation of root growth and nitrogen utilization efficiency [20]. The phenotypes of the *SiMYB3* overexpressing line and the *TAR2* overexpressing line were similar under low-nitrogen stress conditions (Figure 5B–E). The EMSA experiments demonstrate that SiMYB3 can bind to the promoter region of auxin biosynthesis-related genes (Figure 6). Therefore, we hypothesize that SiMYB3 can enhance plant tolerance to low-nitrogen stress by regulating the expression of auxin biosynthesis-related genes and thereby affecting root growth. We also found that a key nitrate transporter, *NRT1.1,* was induced in *SiMYB3* transgenic plants. NRT1.1 (CHL1/NPF6.3) is an influx carrier participating in the root uptake of NO_3_^−^ [52,53]. The role of NRT1.1 as a NO_3_^−^ sensor is versatile because it activates different physiological or developmental responses to NO_3_^−^ via several independent sensing/signaling mechanisms [54]. These results suggest that *SiMYB3* also regulates nitrate transport, which affects the tolerance of transgenic plants to low nitrogen levels.

## 4. Materials and Methods

### 4.1. Swiss-Prot, GO, and KEGG Pathway Annotation

We performed Swiss-Prot function annotation analysis based on the UniProtKB/Swiss-Prot database (http://www.uniprot.org/), GO function annotation analysis based on the GO database (http://geneontology.org/page/go-database), and KEGG pathway annotation analysis based on the KEGG database (http://www.kegg.jp/kegg/ko.html).

### 4.2. Gene Structure and cis-Acting Elements

The TAR2 promoter was evaluated using “Promoter 2.0 Prediction Server” (Promoter 2.0 is available as a web server at http://www.cbs.dtu.dk/services/promoter/) [55]. *Cis*-acting elements were analyzed using the plant *cis-*acting element database Plant Care [56].

### 4.3. Plant Materials and Growth Conditions

*Arabidopsis thaliana* ecotype Columbia (Col-0) was used in this study. *myb9* (SALK_149765C) was obtained from the Arabidopsis Biological Resource Center (ABRC). Professor Yiping Tong (Institute of Genetics and Developmental Biology, Chinese Academy of Sciences) provided the seeds of *TAR2*-OE Arabidopsis. The vernalized seeds were plated on MS plates containing 1% agar and 1% sucrose. The plates were oriented vertically for the seed germination and plant growth stages in growth chambers set at 22 °C, under a 16 h light/8 h dark cycle [20]. To generate *SiMYB3*-OE *Arabidopsis* plants, we introduced the coding region of *SiMYB3* into pBI121, a plant transformation vector, which was controlled by the CaMV35S promoter [57]. The constructs were confirmed by sequencing and then transformed into wild-type plants (Col-0) by the vacuum infiltration method [58]. The transgenic rice seedlings were held at 30 °C under a 14 h light/10 h dark cycle. For rice transformation, *SiMYB3* gene was inserted into pCambia1390 vector and *SiMYB3* controlled by UBI promoter.

### 4.4. Nitrogen Treatments on Media

High N media (3 mM NH_4_NO_3_) and low N media (0.1 mM NH_4_NO_3_) with 1% sucrose, 1% agar, pH 5.8, supplemented with 4 mM CaCl_2_, 1 mM MgSO_4_, 1.5 mM KH_2_PO_4_, 1.5 mM KH_2_PO_4_, 2 mM K_2_SO_4_, 40 μM Na_2_Fe-EDTA, 60 μM H_3_BO_3_, 14 μM MnSO_4_, 1 μM ZnSO_4_, 0.3 μM NaMoO_4_, 0.6 μM CuSO_4_, 0.4 μM NiCl_2_, and 20 nM CoCl_2_ were prepared. Four-day-old seedlings of *Arabidopsis* or surface-sterilized rice were transferred to either high N media or low N media for the stated time periods [20].

### 4.5. Field Low-Nitrogen Stress Testing of Transgenic Rice

To investigate the application potential of *SiMYB3*, field-tests of T3 generation *SiMYB3*-OE plants were performed in paddy fields under normal growth conditions during 2017 and 2018. In order to create low-nitrogen field conditions, we chose red soil regions with poor nutrition at the Rice Research Institute, Jiangxi Academy of Agricultural Sciences (Nanchang, China). In order to deplete the available nutrients in the soil, we planted a generation of conventional rice varieties prior to conducting our field experiment. We also measured the soil nutrient content, and the results showed that the nitrogen content in the low-nitrogen treatment field was 36.2 mg/kg, which was lower than that of normal treatment fields (120.0 mg/kg). To fertilize the field, we used nitrogen 1.8 kg, P_2_O_5_ 0.8 kg, and K_2_O 1.2 kg for every 100 m^2^ of field under normal treatment. No nitrogen with similar levels of nutritional content was used for low-nitrogen treatment. Between the two years of experiments, we planted rice to further deplete the nitrogen levels in the soil. This rice was usually planted in March and transplanted in April of each year.

### 4.6. Subcellular Localization

The ORF of SiMYB3 was cloned into the p16318hGFP vector and fused with the GFP reporter gene under the control of the cauliflower mosaic virus (CaMV) 35S promoter. The protoplast transformation was performed using the method described by Asai et al. [59]. The protoplasts were then viewed with a Zeiss LSM 710 NLO laser scanning microscope (Zeiss, Oberkochen, Germany, http://corporate.zeiss.com) with a 488- or 543-nm laser.

### 4.7. RNA Isolation and Quantitative Real-Time RT-PCR

Total RNA was extracted from seedlings using the Total RNA Extraction Kit (TIANGEN, China). The cDNA was synthesized according to the instructions of the Fast Quant RT Super Mix Reverse Transcription Kit (TransGene, Beijing China). Real-time PCR amplification was performed using a Real Master Mix (SYBR Green, Beijing China) kit (TransGene) and a fluorescence quantitative PCR instrument (ABI7500, USA). The relative expression of the gene in different samples was calculated using the 2^−ΔΔCt^ method, according to the Ct value at the specific fluorescence threshold for each sample.

### 4.8. Protein Purification and Electrophoretic Mobility Shift Assays (EMSA)

The ORF of SiMYB3 was fused in-frame with GST in p4T-1 and expressed in *E. coli* DE3, and purified by standard procedures using glutathione agarose beads (GE Healthcare, Pittsburgh, PA, USA). Briefly, 5 mL of DE3 cells grown overnight and expressing the desired constructs were transferred into 500 mL of LB and grown at 37 °C for 3 h (OD = 1.0). Isopropyl-β-d-thiogalactopyranoside (IPTG, 1 mM) was then added to the media and incubated overnight at 16 °C to induce protein expression. The bacterial cells were sonicated in PBS with 1% Triton X-100 and centrifuged at 10,000 g for 10 min to remove insoluble cell debris. The supernatant was incubated with PBS pre-equilibrated with lutathione agarose beads and rotated at 4 °C for 4 h. After washing five times with PBS, GST-tagged protein was eluted using 10 mM glutathione. For EMSA, 30 ng of purified GST-SiMYB3 recombinant protein, 400 fmol of biotin-labeled annealed oligonucleotides, 2 μL of 10× binding buffer (100 mM Tris, 500 mM KCL, and 10 mM DTT, pH 7.5), 1 μL of 50% (*v*/*v*) glycerol, 1 μL of 100 mM MgCl2, 1 μL of 1 μg/μL poly (dI-dC), and 1 μL of 1% (*v*/*v*) NP–40 were combined and double-distilled water was added to a final volume of 20 μL. Biotin-labeled DNA was detected using the LightShift Chemiluminescent EMSA kit (Thermo Scientific, 20148, Waltham, MA, USA).

## 5. Conclusions

In this study, the transcriptome of foxtail millet under low nitrogen stress was systematically analyzed. The analysis of gene function showed that *Arabidopsis* overexpressing *SiMYB3* had higher root length than WT under low nitrogen stress, which was similar to the phenotype in plants overexpressing TAR2, an auxin synthesis-related gene in *Arabidopsis*. These results suggested that SiMYB3 can regulate root development by regulating plant root auxin synthesis under low nitrogen conditions.

## Data Availability

The transcriptome data is available in the Sequence Read Archive (SRA) under accession number PRJNA516031. All the supporting data are included as additional files.

## Supplementary Materials

Supplementary materials can be found at https://www.mdpi.com/1422-0067/20/22/5741/s1. Figure S1. GO terms and KEGG analysis of differentially expressed genes. The GO biological functions of different expression levels of genes were analyzed by Gene Ontology (Available online: http://www.geneontology.org/). We conducted KEGG Pathway and enrichment analyses of these genes using the Kyoto Encyclopedia of Genes and Genomes (KEGG) Pathway (Available online: http://www.kegg.jp/). Figure S2. The expression of SiMYB3 in the Arabidopsis or rice transgenic lines. RT-PCR analysis showing the expression of SiMYB3 in transgenic Arabidopsis OE1 and OE2 or transgenic rice OE4, OE15, OE28, and OE33. Total RNAs isolated from WT (Col-0), Arabidopsis OE1 and OE2 or CK (kitaake), OE4, OE15, OE28, and OE33 were subjected to RT-PCR analysis using the primers. The Actin genes of Arabidopsis and rice were used as controls. Figure S3. (A) Differential expression of the SiTAR2 gene in foxtail millet under low-nitrogen treatment. (B) Schematic diagram of SiTAR2 promoter region showing the MYB binding motifs. (C) Electrophoretic mobility shift assays (EMSA) indicating SiMYB3 binding specific MYB-like motifs. Supplementary Table S1. Genes induced and repressed at 1 h after SA treatment. Supplementary Table S2. Biological process GO terms of differentially expressed genes. Supplementary Table S3. Cell component GO terms of differentially expressed genes. Supplementary Table S4. Molecular function GO terms of differentially expressed genes. Supplementary Table S5. Twenty-five transcription factors named SiMYB1-25 were significantly differentially expressed genes obtained from the RNA-seq analysis. Supplementary Table S6. List of the primers used in PCR analyses.

## Abbreviations

GO	Gene Ontology
bp	Base Pair
cDNA	Complementary DNA
CK	Control Check
CDS	Coding Sequence
RT-PCR	Reverse Transcription-Polymerase Chain Reaction
WT	Wild Type
OE	Overexpression Line
LR	Lateral Root
LN	Low Nitrogen
LP	Low Phosphorus
LK	Low Potassium
TF	Transcription Factor
