# Supplementary figures and images for: SiMYB3 in Foxtail Millet (Setaria italica) Confers Tolerance to Low-Nitrogen Stress by Regulating Root Growth in Transgenic Plants
Source: Int J Mol Sci. 2019 Nov 15;20(22):5741. doi: 10.3390/ijms20225741 (PMC6888739; doi:10.3390/ijms20225741)

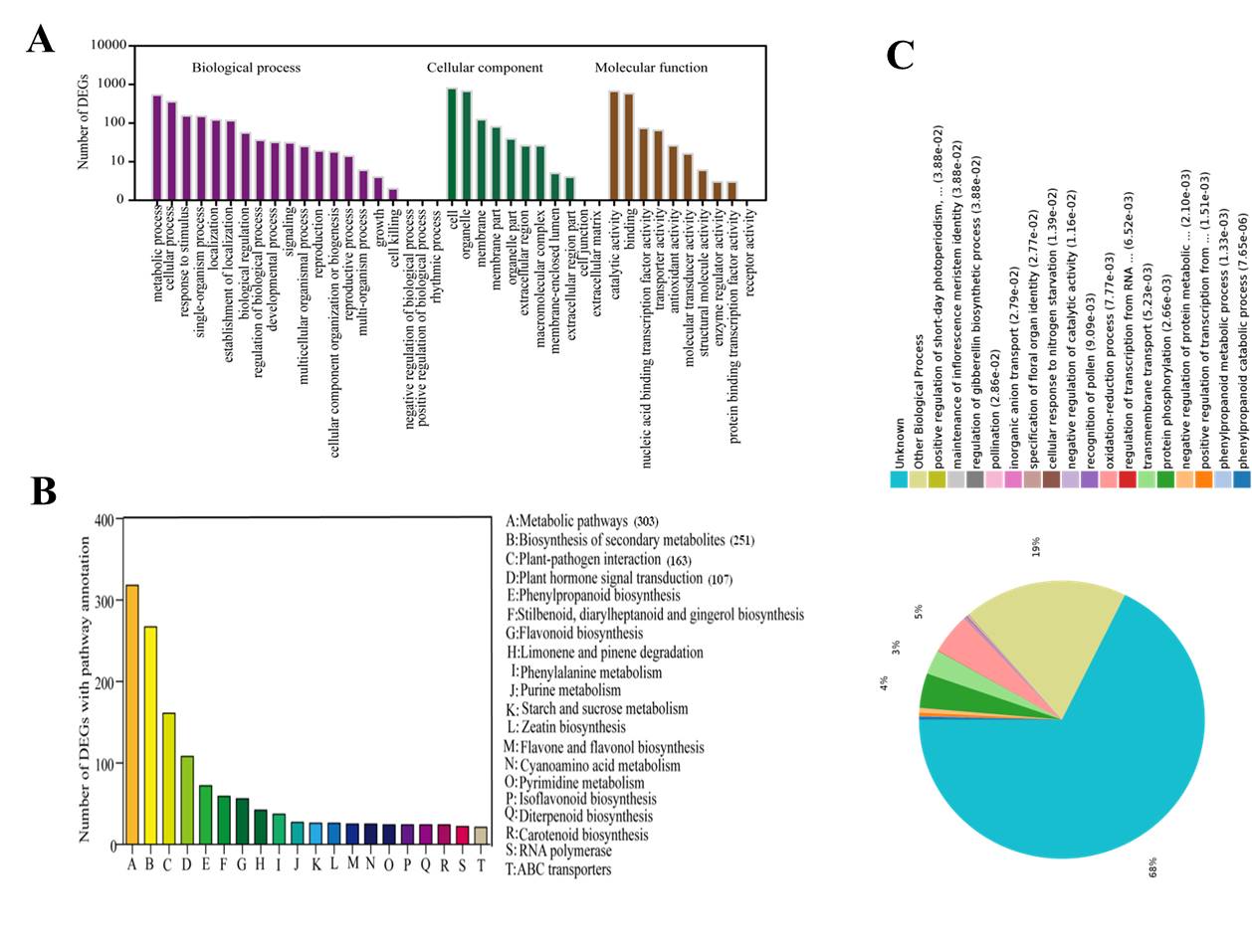

Supplement: Supplementary file 1 [file ijms-20-05741-s001.zip › Supplementary files/Supplementary fig. 1.jpg]

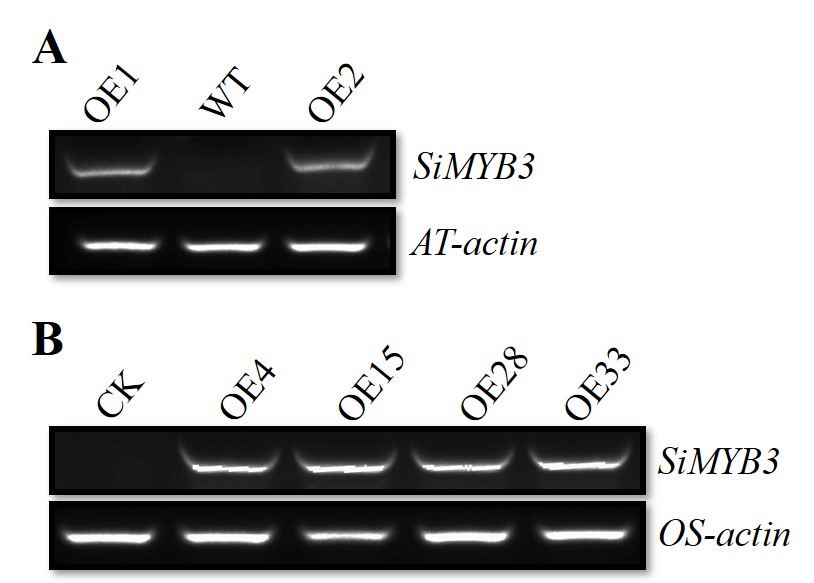

Supplement: Supplementary file 1 [file ijms-20-05741-s001.zip › Supplementary files/Supplementary fig. 2.jpg]

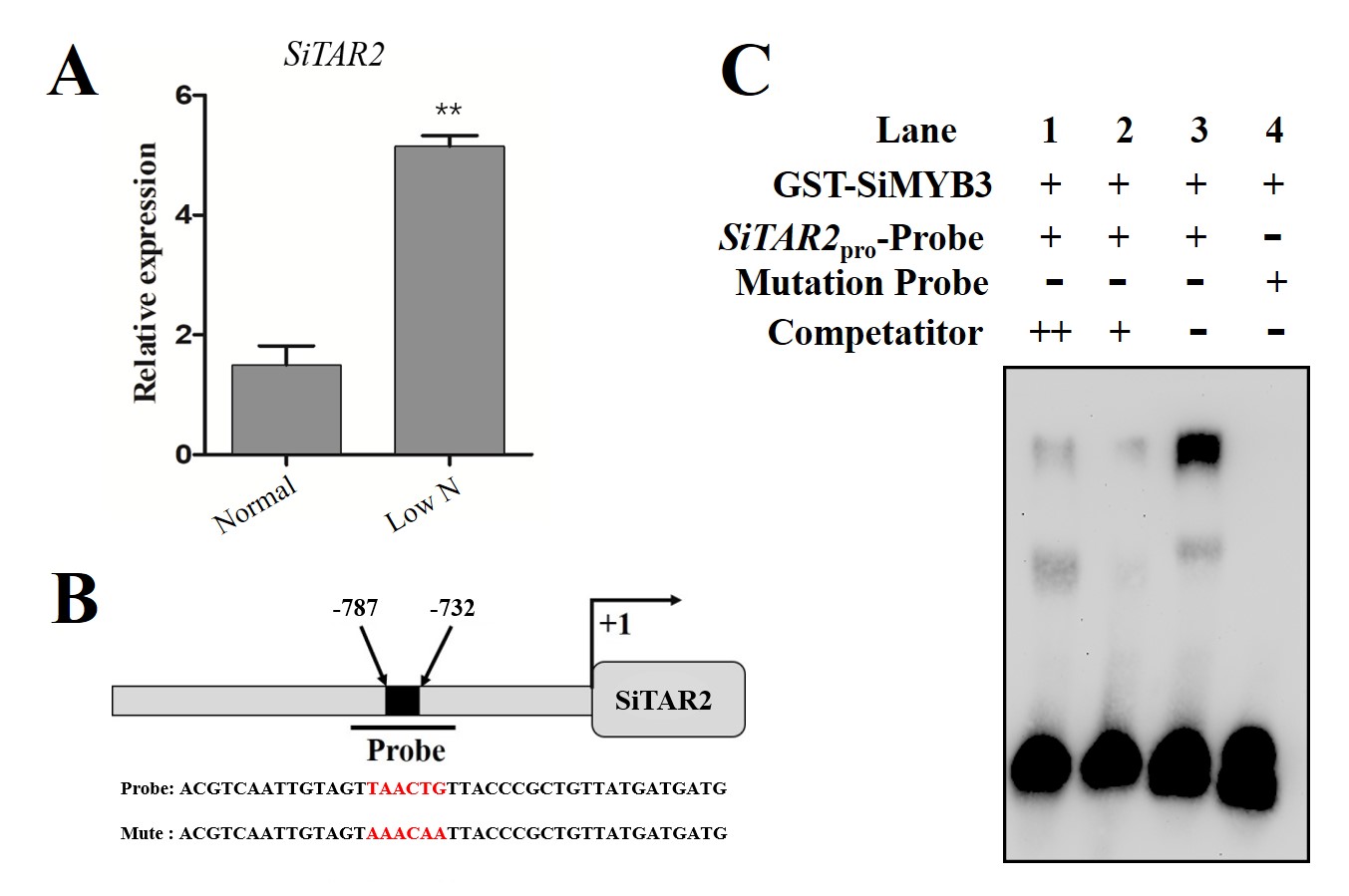

Supplement: Supplementary file 1 [file ijms-20-05741-s001.zip › Supplementary files/Supplementary fig. 3.jpg]
