# Supplementary material for: SiMYB3 in Foxtail Millet (Setaria italica) Confers Tolerance to Low-Nitrogen Stress by Regulating Root Growth in Transgenic Plants
Source: Int J Mol Sci. 2019 Nov 15;20(22):5741. doi: 10.3390/ijms20225741 (PMC6888739; doi:10.3390/ijms20225741)
Supplement: Supplementary file 1 [file ijms-20-05741-s001.zip › Supplementary files/Supplemantary table 5.doc]

Supplementary table 5. The genes characteristics of 25 MYB family in foxtail millet

| **Gene name** | **Gene ID** | Used name[1] | **Amino acid** | **Molecular mass (kD)** | **pI** | **Gene length (bp)** | **Exon count** | **Chromosome** |
| --- | --- | --- | --- | --- | --- | --- | --- | --- |
| *SiMYB1* | Si032544m.g | SiMYB037 | 226 | 24.91 | 5.77 | 774 | 2 | 2 |
| *SiMYB2* | Si002888m.g | SiMYB121 | 214 | 23.54 | 9.03 | 2128 | 2 | 5 |
| *SiMYB3* | Si012660m.g | SiMYB167 | 315 | 34.17 | 5.79 | 1121 | 2 | 7 |
| *SiMYB4* | Si011791m.g | SiMYB210 | 235 | 26.19 | 9.42 | 1398 | 2 | 7 |
| *SiMYB5* | Si026524m.g | SiMYB176 | 368 | 40.03 | 6.31 | 1805 | 5 | 8 |
| *SiMYB6* | Si010384m.g | SiMYB168 | 372 | 39.45 | 5.06 | 1320 | 3 | 7 |
| *SiMYB7* | Si031939m.g | SiMYB038 | 278 | 30.80 | 5.07 | 935 | 2 | 2 |
| *SiMYB8* | Si010794m.g | SiMYB169 | 278 | 30.74 | 9.07 | 2403 | 6 | 7 |
| *SiMYB9* | Si014183m.g | SiMYB151 | 290 | 30.87 | 9.2 | 1539 | 3 | 6 |
| *SiMYB10* | Si026803m.g | SiMYB180 | 237 | 27.21 | 8.84 | 1422 | 3 | 8 |
| *SiMYB11* | Si022874m.g | SiMYB066 | 286 | 30.46 | 8.71 | 1542 | 3 | 3 |
| *SiMYB12* | Si026734m.g | SiMYB177 | 280 | 31.11 | 8.63 | 1487 | 3 | 8 |
| *SiMYB13* | Si022816m.g | SiMYB071 | 297 | 32.69 | 6.31 | 1617 | 2 | 3 |
| *SiMYB14* | Si030750m.g | SiMYB041 | 278 | 30.38 | 5.41 | 4764 | 2 | 2 |
| *SiMYB15* | Si018049m.g | SiMYB020 | 275 | 30.18 | 8.4 | 2754 | 6 | 1 |
| *SiMYB16* | Si022265m.g | SiMYB069 | 393 | 43.60 | 6.39 | 1934 | 6 | 3 |
| *SiMYB17* | Si013994m.g | SiMYB149 | 357 | 39.55 | 5.78 | 3789 | 3 | 6 |
| *SiMYB18* | Si007179m.g | SiMYB097 | 242 | 27.08 | 7.08 | 2666 | 5 | 4 |
| *SiMYB19* | Si030152m.g | SiMYB044 | 382 | 40.31 | 6.3 | 1354 | 3 | 2 |
| *SiMYB20* | Si016955m.g | SiMYB002 | 507 | 56.01 | 5.94 | 2093 | 6 | 1 |
| *SiMYB21* | Si036412m.g | SiMYB002 | 349 | 37.38 | 6.35 | 2050 | 5 | 9 |
| *SiMYB22* | Si014685m.g | SiMYB140 | 116 | 13.61 | 8.62 | 1619 | 3 | 6 |
| *SiMYB23* | Si017415m.g | SiMYB023 | 400 | 41.81 | 6.86 | 2516 | 3 | 1 |
| *SiMYB24* | Si018182m.g | SiMYB013 | 249 | 28.16 | 5.91 | 1324 | 2 | 1 |
| *SiMYB25* | Si002653m.g | SiMYB139 | 253 | 27.13 | 6.48 | 1297 | 1 | 5 |

[1]

1.	Muthamilarasan M, Khandelwal R, Yadav CB, Bonthala VS, Khan Y, Prasad M**: Identification and molecular characterization of MYB Transcription Factor Superfamily in C4 model plant foxtail millet (Setaria italica L**.)*. PLoS On*e 2014**,** 9(10):e109920.
