# Supplementary material for: SiMYB3 in Foxtail Millet (Setaria italica) Confers Tolerance to Low-Nitrogen Stress by Regulating Root Growth in Transgenic Plants
Source: Int J Mol Sci. 2019 Nov 15;20(22):5741. doi: 10.3390/ijms20225741 (PMC6888739; doi:10.3390/ijms20225741)
Supplement: Supplementary file 1 [file ijms-20-05741-s001.zip › Supplementary files/Supplemantary table 6.doc]

Supplementary table 6. List of the primers used in this research.

| **Primer name** | **Primer sequence（5′–3′）** |
| --- | --- |
| *SiMYB3-F* | ATGGGGAGGTCGCCGTGCTGCT |
| *SiMYB3-R* | TCATAGTGGCAAGCTGCTGCTCTC |
| *SiMYB3-pBI121-F* | TAGAGGATCCCCGGGATGGGGAGGTCGCCGT |
| *SiMYB3-pBI121-R* | AGTGGATCCCCCGGGTCATAGTGGCAAGCTG |
| *SiMYB3-GFP-F* | TATCTCTAGAGGATCCATGGGGAGGTCGCCGT |
| *SiMYB3-GFP-R* | TGCTCACCATGGATCCTAGTGGCAAGCTGCTG |
| *SiMYB3-RT-F* | CATGGCCAATAATAACTTCGGC |
| *SiMYB3-RT-R* | TTCATCTGGTCCTGGAAGTTTG |
| *SiActin-F* | GGCAAACAGGGAGAAGATGA |
| *SiActin-R* | GAGGTTGTCGGTAAGGTCACG |
| *HYG*-ddpcr-P | 5' FAM-AAGCACGAGATTCTTCGCCC-BHQ1 3' |
| *HYG*-ddpcr-F | GAAAAAGCCTGAACTCACCG |
| *HYG*-ddpcr-R | CATATCCACGCCCTCCTAC |
| 121tair1 | ATTGAATCCTGTTGCCGGTCTT |
| 121tair2 | GTAATGCATGACGTTATTTATGAGATG |
| 121tair3 | GCGCGCAAACTAGGATAAATTATC |
| FP1 | GTAATACGACTCACTATAGGGCACGCGTGGTNTCGASTWTSGWGTT |
| 1390-tair1 | ACTTCAGCCTGCCGGTACCGCC |
| 1390 -tair2 | GTCCTGCCCGTCACCGAGATTTGA |
| 1390-tair3 | TATTCAGTAATCTCGGCCAATATCC |
| FSP1 | GTAATACGACTCACTATAGGGC |
| FSP2 | ACTATAGGGCACGCGTGGT |
